# An fMRI approach to assess intracranial arterial-to-venous cardiac pulse delay in aging

**DOI:** 10.1162/IMAG.a.969

**Published:** 2025-10-30

**Authors:** Adam M. Wright, Tianyin Xu, John Koo, Yi Zhao, Yunjie Tong, Qiuting Wen

**Affiliations:** Department of Radiology and Imaging Sciences, Indiana University School of Medicine, Indianapolis, IN, United States; Weldon School of Biomedical Engineering Department, Purdue University, West Lafayette, IN, United States; Department of Biostatistics and Health Data Science, Indiana University School of Medicine, Indianapolis, IN, United States

**Keywords:** arterial-venous pulse delay (A-V delay), cardiac pulsatility, intracranial compliance, functional MRI, aging

## Abstract

Each heartbeat generates a cardiac pressure wave that propagates through the brain and travels from large arteries through cerebrospinal fluid and brain tissue, compressing the venous sinuses and producing venous blood pulsatility. The delay between arterial and venous pulsation (A-V delay) is an insightful marker of intracranial compliance and the intracranial mechanical environment. We developed a novel approach to extract A-V delay from conventional resting-state functional MRI (fMRI) scans, leveraging fMRI’s sensitivity to vessel pulsations in large cerebral arteries and the superior sagittal sinus (SSS). This fully automated method was applied to the Human Connectome Project – Aging dataset to analyze 578 participants aged 35 to 90 years. The mean A-V delay was 78 ± 32 msec; it shortened by 4 msec for every decade of aging and was 12 msec faster in men than women, highlighting age-related and sex-specific differences. We also identified a within-SSS pattern of pulsations, characterized by an earlier posterior pulsation and a later anterior pulsation. This pattern opposes the direction of blood flow, supporting that the SSS is passively compressed and tied to a distinct intracranial pulse transmission. Overall, this work demonstrates the feasibility of extracting an fMRI-based A-V delay, uncovering a previously unexplored capability of fMRI. This approach broadens the potential applications of fMRI by adding a biomechanical dimension to fMRI’s established roles in evaluating neuronal and hemodynamic function. Given the widespread availability of fMRI, this approach can be applied in future studies to investigate biomechanical changes in various disease conditions.

## 1 Introduction

After every heartbeat, a powerful pulsation of blood enters the skull, resulting in a small and short-lived increase in intracranial fluid that is quickly balanced by the outflow of cerebrospinal fluid (CSF) and venous blood (Alperin, 2020; Greitz et al., 1992; Wagshul et al., 2011). The rigidity of the skull and incompressibility of CSF creates a unique intracranial environment where arterial pulsations are transmitted through the CSF and mechanically compress the cerebral venous system (see (Wagshul et al., 2011) for review). The resulting venous blood pulsatility is a distinct brain feature (Bateman, 2002; Driver et al., 2020). Importantly, the pressure wave compressing the venous system originates from the cerebral arteries and is dispersed through the CSF, independent of the blood circulation pathway. The time delay between arterial and venous pulsation (A-V delay) is sensitive to mechanical changes in the intracranial space and provides a non-invasive assessment of intracranial compliance (Bateman, 2000, 2004). As intracranial compliance decreases, stiffer mechanical properties increase propagation speeds, thereby reducing the A-V delay, making it a promising measure to investigate intracranial biomechanical changes.

Despite its relevance, the current assessment of A-V delay remains limited to phase-contrast MRI. PC MRI quantifies the A-V delay by capturing arterial and venous flow waveforms and calculating the time delay between them. Reductions in A-V delay have been reported in normal pressure hydrocephalus (Bateman, 2000, 2002; Miyati et al., 2007) and Alzheimer’s disease (Bateman, 2004; Rivera-Rivera et al., 2021), demonstrating its sensitivity to intracranial mechanical changes. However, PC-MRI is generally not included in routine MRI protocols, which limits the study of A-V delay across broader clinical populations.

Functional MRI (fMRI), widely recognized for its sensitivity to the blood oxygen level-dependent (BOLD) effect in cortical regions, also detects flow effects in larger cerebral arteries and venous sinuses. Previous studies have demonstrated consistent pulsatile signals from large cerebral arterial and venous blood in cardiac-aligned fMRI, where cardiac systole results in a decreased fMRI signal due to fast flow and spin dephasing (Aslan et al., 2019; Hermes et al., 2023; Rajna et al., 2021; Tong et al., 2014). Recently, our group leveraged these unique fMRI signal fluctuations to investigate their coupling with paravascular cerebrospinal fluid (CSF) dynamics (Wright, Wu, et al., 2024) and developed a data-driven approach to segment large cerebral arteries and venous sinuses (Wright, Xu, et al., 2024). We observed robust cardiac pulsations across different datasets (Nair et al., 2025; Wright et al., 2025). Building on fMRI’s established sensitivity to vessel pulsations, we hypothesize these signals can be further utilized to measure the A-V delay. This approach could provide a widely accessible method to assess intracranial biomechanical properties in larger and more diverse clinical populations.

This study proposed a novel, fully automated analytical approach for extracting A-V pulse delay using resting-state fMRI, revealing a previously unexplored capability of fMRI. The approach was applied to the large-scale, publicly available Human Connectome Project-Aging (HCP-A) dataset to investigate the impact of healthy aging and biological sex on A-V delay measurements. This method enabled the generation of A-V delay maps, where the spatial pattern was examined across different age groups. The reproducibility of the A-V delay measurements was evaluated using repeated fMRI scans from the HCP-A dataset.

## 2 Methods

### 2.1 Human participants

This study used participant data from two cohorts: the Human Connectome Project – Aging (HCP-A) cohort to characterize the A-V delay changes associated with age, biological sex, and various covariates, and a local cohort with a fast fMRI scan to validate the fMRI A-V delay methodology. Both cohorts included adults with typical health status and no history of stroke or clinical dementia. HCP-A data used in this work included structural MRI, resting-state fMRI, participant demographics, ambulatory health measures, blood measures, and cognitive scoring, as outlined in (Bookheimer et al., 2019). All HCP-A participants provided written informed consent as outlined in (Bookheimer et al., 2019; Harms et al., 2018). The local data used in this work included structural MRI, resting-state fMRI, and participant demographics. All local cohort participants provided written informed consent for the study, which the Indiana University Institutional Review Board approved.

### 2.2 Imaging acquisition

The HCP-A cohort was imaged with a 3 Tesla Prisma Siemens scanner using a 32-channel head-neck coil. This analysis utilized a T1-weighted (T1-w) anatomical scan and a pair of resting-state fMRI scans. The T1-w scan used a 3D magnetization rapid gradient echo (MPRAGE) sequence with a voxel size = 0.8 × 0.8 × 0.8 mm³. The fMRI was acquired with the following parameters: repetition time = 800 msec, echo time = 37.0 msec, flip angle = 52º, voxel size = 2.0 × 2.0 × 2.0 mm³, volumes = 488, multiband (MB) factor = 8, anterior-posterior (A-P) phase-encoding, and an acquisition time = 6 min and 30 sec. The repeated fMRI scans were completed over two separate imaging sessions in 1 day or across 2 days and were used in the reproducibility assessment. Simultaneous finger photoplethysmography (PPG) was acquired during fMRI scans with a sampling rate of 400 Hz. During HCP-A fMRI scans, participants were instructed to stay awake with their eyes open and blink normally while looking at a fixed white crosshair on a black background (Harms et al., 2018).

The local cohort was imaged with a 3 Tesla Prisma Siemens scanner using a 64-channel head-neck coil. The T1-w scan used a 3D MPRAGE sequence with a voxel size = 1.0 × 1.0 × 1.0 mm³. The fMRI was acquired with the following parameters: repetition time = 363 msec, echo time = 30 msec, flip angle = 35º, voxel size = 2.5 × 2.5 × 2.5 mm³, volumes = 500, MB factor = 8, A-P phase-encoding, an acquisition time = 3 min and 2 sec, and with simultaneous finger PPG acquired at 400 Hz. A single fMRI scan was completed, and the participants were instructed to stay awake with their eyes open.

### 2.3 Image processing

#### 2.3.1 fMRI preprocessing

The first ten fMRI volumes were removed to reduce T1-relaxation effects. In the HCP-A and local cohorts, 478 and 490 time points were used in post-processing, respectively. Motion parameters were measured to monitor scan quality (FSL: mcflirt). Motion and slice time corrections were not applied to retain real-time slice acquisitions for further processing with the simultaneous finger PPG.

#### 2.3.2 Cerebral artery and superior sagittal sinus segmentation

A recently developed automatic data-driven algorithm was used to segment the large cerebral arteries and superior sagittal sinus (SSS) in fMRI space by leveraging the pulsatile and reproducible cardiac-induced signal fluctuations in large cerebral arteries and SSS (Wright, Xu, et al., 2024). The arterial segmentation included the anterior, middle, and posterior cerebral arteries (ACA, MCA, PCA).

#### 2.3.3 Arterial-venous cardiac pulse delay calculation using large vessel fMRI signal dynamics

Large vessel fMRI signal is sensitive to changes in blood flow velocity across the cardiac cycle. During the systolic phase, the cardiac pulse increases blood flow velocities in both cerebral arteries and large veins. These high blood velocities result in MR spin-spin dephasing (Von Schulthess & Higgins, 1985), decreasing the fMRI signal. This signal dependence has been observed by multiple studies, consistently finding that fMRI signal minima occur during cardiac systole in both cerebral arteries and the SSS (Hermes et al., 2023; Rajna et al., 2021; Wright, Wu, et al., 2024). The similar trend in blood flow velocities and corresponding fMRI signal changes in arteries and veins can be leveraged to measure the cerebral A-V pulse delay in an approach outlined below.

**Fig. 1.**
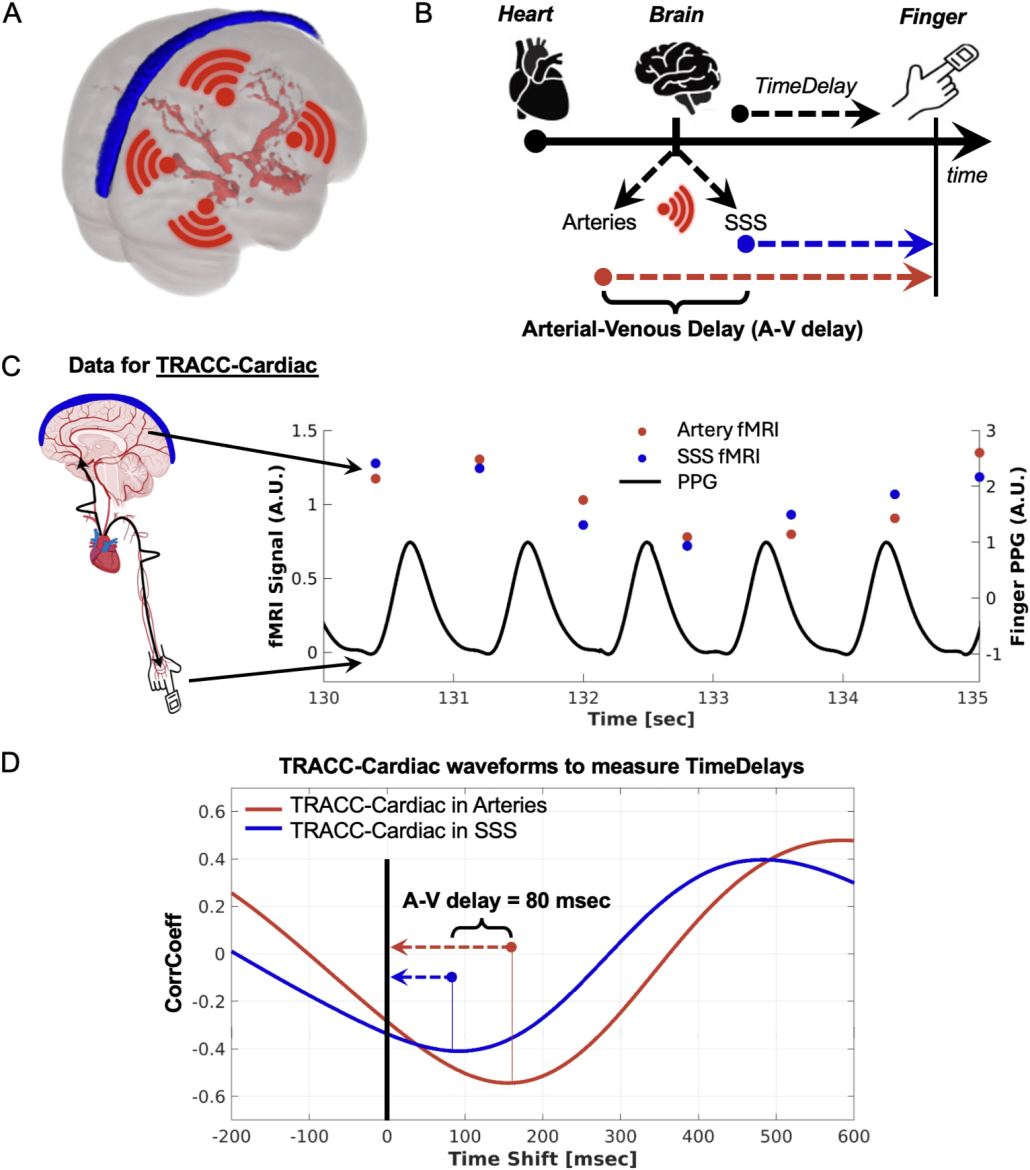
Summary of the fMRI-based approach to calculate arterial-venous (A-V) delay. (A) Following each heartbeat, a cardiac impulse propagates across the brain from large cerebral arteries to large cerebral veins. (B) The A-V delay was calculated as the time difference between the cardiac pulse arrival in the cerebral arteries and the superior sagittal sinus (SSS) using the finger pulse arrival as a reference (TimeDelay). (C) A representative participant’s vascular fMRI and finger PPG signals were used to compute the vascular TimeDelay with the TRACC-Cardiac approach. (D) The A-V delay derived from the vascular TimeDelays, corresponding to the maximum CorrCoeff in TRACC-Cardiac waveforms. These waveforms were generated by time-aligned cross-correlation of artery voxels (red) and SSS voxels (blue) fMRI signals with the finger PPG shown in C. Abbreviations: A-V delay – arterial-venous delay, SSS – superior sagittal sinus, PPG – finger photoplethysmography, CorrCoeff – cross-correlation coefficient.

Conceptually, the fMRI-based A-V delay was calculated in two steps (Fig. 1). First, the difference in cardiac pulse arrival times between the brain and finger was determined and referred to as the TimeDelay, assessed using a technique termed TRACC-Cardiac as described in Wright et al. (2025) (preprint) and implemented in Wen et al. (2023, 2025), detailed below. Second, the A-V delay was calculated as the difference in the mean TimeDelay between the arterial and SSS regions of interest. Figure 1B shows the cardiac pulse propagation timeline to the arteries, SSS, and finger, demonstrating that the A-V delay equals the TimeDelay difference between the arteries and the SSS (a more in-depth visual derivation is provided in Supplemental Fig. 1). The TimeDelay measurements across the ACA, MCA, and PCA were consistent (Supplemental Fig. 2), so their average TimeDelay was used. The specific signal processing steps to compute the voxel-wise TimeDelay and A-V delay are summarized below.

The TimeDelay was measured using the TRACC-Cardiac method outlined in Wright et al. (2025) (preprint), with voxel-wise fMRI measurements performed as follows: The fMRI time series were filtered using a 4^th^-order high-pass Butterworth filter (cutoff frequency = 0.1 Hz) with a zero-phase shift implementation (Matlab: butter.m and filtfilt.m) to reduce the impact of low-frequency signal fluctuations and then normalized to z-score. The peak PPG amplitudes were set to a fixed range of ±1 to minimize the impact of low-frequency signal fluctuations. The TimeDelay for each voxel was defined as the time shift between the absolute maximum cross-correlation (Peak CorrCoeff) of the fMRI signal and the finger PPG within a window of ±300 msec (empirically set to capture the TimeDelay and minimize computational expense; see Supplemental Fig. 3). The cross-correlation was calculated between the fMRI signal and the time-shifted PPG as shown in Equation 1:

$$CorrCoeff\left(\tau \right)=\;\frac{1}{N-1}\sum_{n=1}^{N}fMRI\left(n\right)\;\cdot \;PPG\left(n+\tau \right)$$ (1)

where CorrCoeff was the correlation coefficient, N was the number of time points in the fMRI signal, and τ represented the time shift that was solved for between ±300 msec in steps of 2.5 msec. The cross-correlation’s temporal resolution is determined by the sampling frequency of the shifted signal; by shifting the PPG signal instead of the fMRI signal, the cross-correlation temporal resolution was maximized to 400 Hz (corresponding to a time-shift step of 2.5 msec). After each 2.5 msec shift, the PPG signal was downsampled to the fMRI time points, enabling the correlation calculation. The TimeDelay was defined as the τ value that maximized the CorrCoeff (Equation 2):

$$TimeDelay=\arg\max_{\tau }|CorrCoeff(\tau )|$$ (2)

Finally, the A-V delay was calculated as the difference in the weighted mean TimeDelay between the arterial and SSS regions of interest, where the CorrCoeff weighted each voxel’s TimeDelay:

$$Arterial-Venous\;\left(A-V\right)\;delay=\;\frac{\sum_{i=1}^{N_{A}}{\left|CorrCoeff\left(\tau \right)\right|}_{i}\cdot \;TimeDelay_{i}}{\sum_{i=1}^{N_{A}}{\left|CorrCoeff\left(\tau \right)\right|}_{i}}-\frac{\sum_{i=1}^{N_{SSS}}{\left|CorrCoeff\left(\tau \right)\right|}_{i}\cdot \;TimeDelay_{i}}{\sum_{i=1}^{N_{SSS}}{\left|CorrCoeff\left(\tau \right)\right|}_{i}}$$ (3)

where |CorrCoeff(τ)| was the weight of the TimeDelay, i was the voxel number, N_A_ was the number of arterial segmentation voxels, and N_SSS_ was the number of SSS segmentation voxels. With the HCP-A fMRI imaging parameters, simulations suggest that the mean errors in TimeDelay measurements in the major cerebral arteries and SSS are approximately 2.8 msec, with the 5^th^–95^th^ percentile error ranging from 0 to 7.5 ms (Wright et al., 2025) (preprint).

#### 2.3.4 Arterial-to-venous delay map in MNI space

Group-mean A-V delay maps were generated in the MNI space to visualize the spatial pattern of the pulse delay and compare it across age groups. Subject-wise maps were first created in the fMRI space by subtracting the mean MCA TimeDelay from the voxel-wise TimeDelay. This step aligned all subjects to the same reference (mean MCA delay = 0 msec), removing the impact of time delay between the MCA and finger. The MCA region was delineated using the statistical brain atlas developed by Dunås et al. (2017). The A-V delay maps were subsequently transformed from fMRI space into MNI152 1 × 1 × 1 mm^3^ space for group analysis using a two-step registration process. First, the fMRI map was transformed into the participant’s T1-weighted space (FSL: FLIRT, linear interpolation, rigid-body registration) and then from T1-weighted to MNI space (ANTs: antsApplyTransforms, nonlinear interpolation, nonrigid body registration).

#### 2.3.5 Brain volume segmentation

T1-weighted anatomical scan parcellations were completed with FreeSurfer 6.0 (recon -all) (Fischl, 2012), sourced directly from HCP-A Lifespan 2.0 processed outputs. The output segmentation volumes were used as input covariates in statistical analysis. The normalized brain volume was determined by the FreeSurfer output BrainSegVol_to_eTIV, which is the brain volume divided by the estimated total intracranial volume (eTIV).

### 2.4 Image quality criteria

fMRI scans were checked for finger PPG quality and scan motion. Finger PPG quality was assessed based on the percentage of signal power centered around the cardiac frequency, as previously published (Wright, Xu, et al., 2024). PPG with less than 80% signal power within ±0.15 Hz of the cardiac fundamental frequency were considered unsuitable for analysis. Scans with absolute motion exceeding 1 mm for more than 15% of the scan length were considered unsuitable for analysis. The absolute motion was measured as the root mean square displacement relative to the middle fMRI volume (FSL: mcflirt). fMRI scans were excluded from the analysis for either poor finger PPG quality or excessive scan motion.

### 2.5 Validation test of fMRI arterial-venous cardiac pulse delay calculation in the presence of cardiac aliasing

The local cohort’s fast fMRI (TR = 0.363 sec) scan was used to test the impact of cardiac aliasing on the fMRI A-V delay calculation. First, we calculated the A-V delay with the native TR = 0.363 sec. We then resampled the scan to use every other volume, resulting in an effective TR (eTR) = 0.726 sec, and recalculated the A-V delay. We then compared the A-V delays between the native TR and eTR in all cases where the cardiac signal was critically sampled at the native TR and became aliased at the eTR.

### 2.6 Statistical analysis

A best-fit linear mixed-effects model assessed the relationship between A-V delay and covariates, including participant demographics, ambulatory measures, blood test results, and segmentation volumes (all variables are outlined in Supplemental Table 1). The model was required to include participant demographics (age, biological sex, race, ethnicity), body mass index (BMI), and heart rate. The initial model included all covariates sequentially removed and added (backward exclusion and forward inclusion) to optimize the Bayesian information criterion for the best fit.

**Table 1.** The best-fit linear mixed-effects model for arterial-venous (A-V) delay.

Variable	β	Standard error	t-value
Age [years]	-0.437	0.107	-4.084***
Biological sex [M vs. W]	-12.056	2.608	-4.622***
HR [bpm]	-0.346	0.109	-3.184**
Normalized brain volume [%]	0.661	0.215	3.073**
SSS volume [cm^3^]	3.358	0.683	4.921***
BMI [$\frac{\text{kg}}{{\text{m}}^{2}}]$	-0.184	0.264	-0.697
Race compared to White			
Black	4.384	3.571	1.228
Asian	3.674	4.997	0.735
American Indian	12.938	21.235	0.609
More than one race	3.805	5.941	0.640
Other	4.795	9.431	0.508
Ethnicity [not Latino vs. Latino]	-0.811	4.576	-0.177

The significant predictors of A-V delay included age, biological sex, heart rate, normalized brain volume, and superior sagittal sinus (SSS) segmentation volume.

**p < 0.01, ***p < 0.001.

Abbreviations: BMI – body mass index, HR – heart rate, SSS – superior sagittal sinus, M – men, W – women, bpm – beats per minute.

The study had repeated scan-specific measures from the repeated fMRI scans, including A-V delay, scan heart rate, arterial segmentation volume, and SSS segmentation volume. Pearson correlation was applied to assess the strength of the linear relationship between repeated measures. Bland-Altman plots were used to visualize the agreement between repeated measures. A paired two-tailed t-test assuming equal variance tested the agreement between repeated measures, with p < 0.05 indicating a significant difference in repeated measures. Measurement reproducibility was also assessed with intraclass correlation coefficients (ICC). p < 0.05 was considered significant for all statistical tests.

## 3 Results

### 3.1 Selection criteria and participant demographics

Only fMRI’s that passed quality checks were included in the analysis. Of the 1414 scans, 432 were excluded: 40 scans exhibited both high motion and poor PPG quality, 116 exclusively had high motion, and 276 exclusively had poor PPG quality. Of the remaining 982 scans, 35 were removed for physiologically implausible measures: 31 scans had an A-V delay of less than 0 msec, and 4 had normalized brain volume estimates exceeding 100%. Ultimately, 947 scans from 578 participants were included in the analysis. Participants were 58.8 ± 14.5 years old (range: 36−90 years), including 325 women and 253 men.

### 3.2 fMRI arterial-venous pulse delay was reduced with age and lower in men

**Fig. 2.**
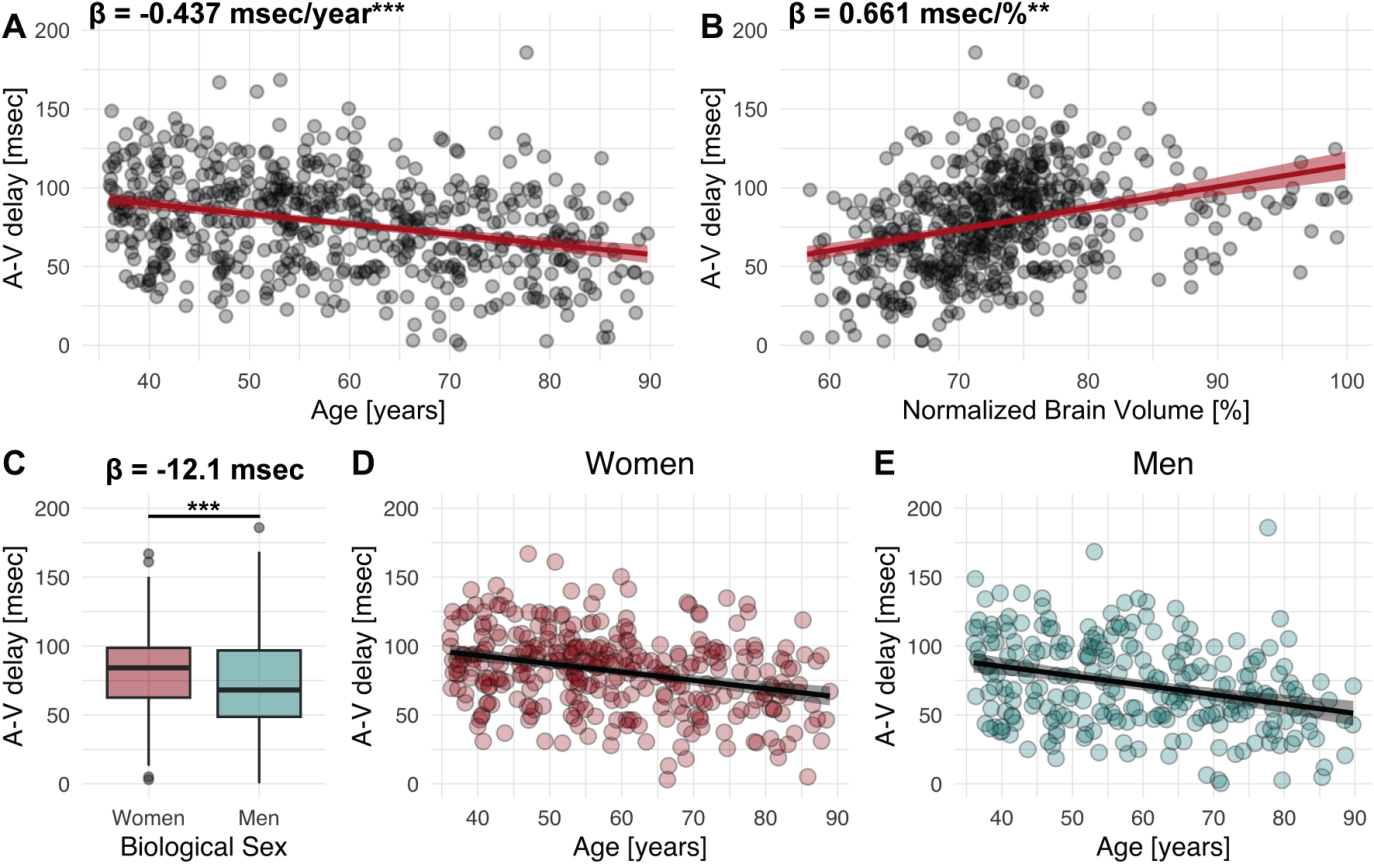
Summary of significant covariates in the arterial-venous (A-V) delay best-fit linear mixed-effects model. (A) A-V delay versus age. (B) A-V delay versus normalized brain volume. (C) A-V delay in women versus men. (D, E). A-V delay versus age stratified by biological sex. **p < 0.01, ***p < 0.001. Abbreviations: A-V delay – arterial-venous delay.

The A-V delay best-fit linear mixed-effects model is summarized in Table 1, and the covariate’s relationship with the A-V delay is illustrated in Figure 2. A-V delay decreased with age (β = -0.437 msec/year, SE = 0.107, p < 0.001), heart rate (β = -0.346 msec/bpm, SE = 0.109, p < 0.01), and was lower in men (β = -12.056, SE = 2.608, p < 0.001). A-V delay increased with normalized brain volume (β = 0.661 msec/% volume, SE = 0.215, p < 0.01) and SSS segmentation volume (β = 3.358 msec/cm^3^, SE = 0.683, p < 0.001). The relationship between A-V delay and vessel segmentation volumes is summarized in Supplemental Figure 4. The covariates, including ambulatory and blood test measures, did not improve the model. A post hoc analysis determined that the interaction between age and biological sex did not improve the model fit or have a significant relationship with A-V delay (see Supplemental Results).

### 3.3 The superior sagittal sinus arterial-venous delay progressed anteriorly

**Fig. 3.**
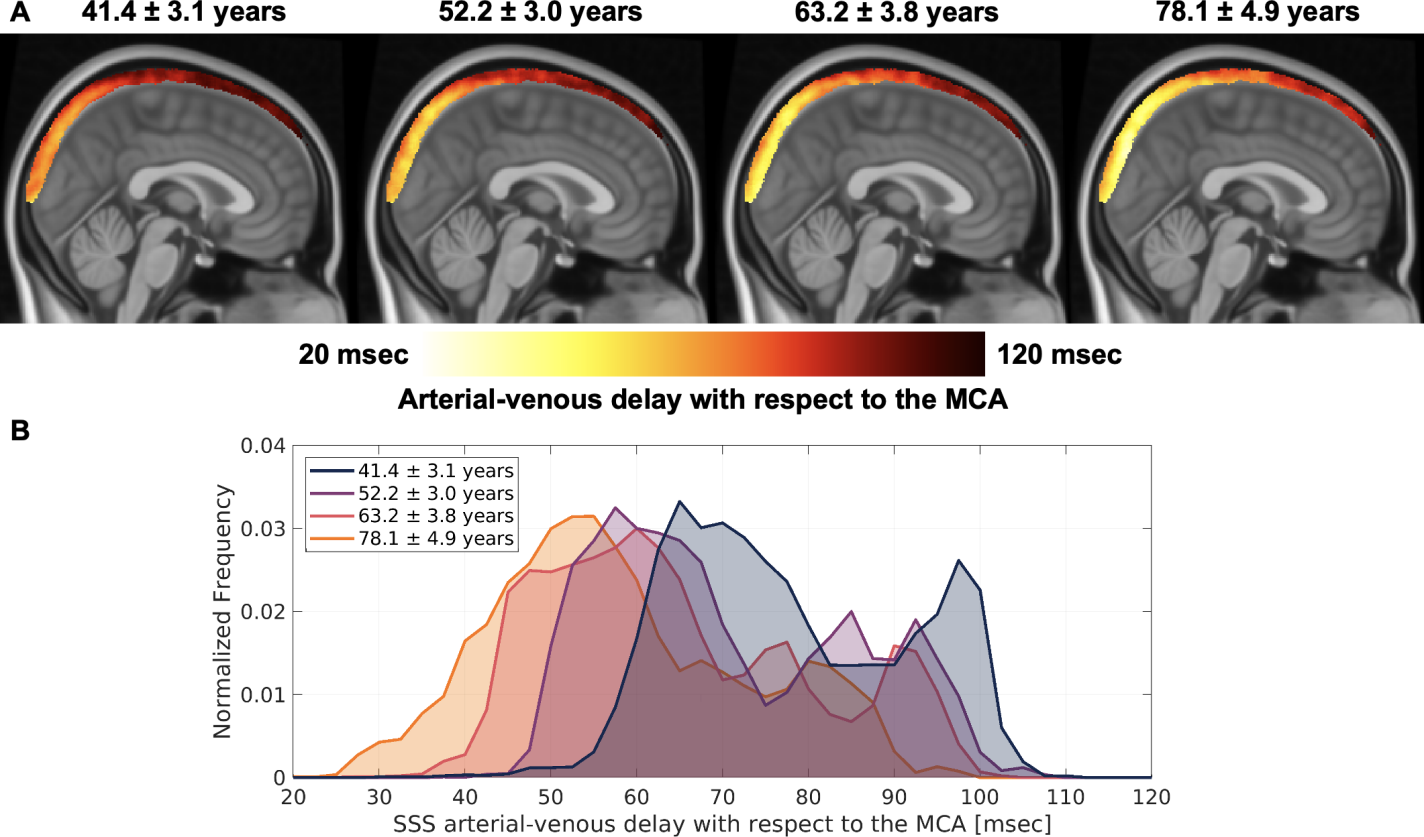
The arterial-venous (A-V) delay in the superior sagittal sinus (SSS) with respect to the middle cerebral artery (MCA). (A) Average A-V delay maps in the Montreal Neurological Institute 1 mm T1 atlas (MNI-152) in four groups with increasing age (mean ± std). The sagittal view is displayed with the x-coordinate = 1. (B) The distribution of voxel-wise A-V delay in the SSS for each age group. The distributions were normalized to have an area under the curve equal to 1, with a bin width = 2.5 msec. Abbreviations: MCA – middle cerebral artery, SSS – superior sagittal sinus.

Four age groups were used to generate group-averaged A-V delay maps in MNI space (group demographics are summarized in Supplemental Table 2). In all age groups, the shortest A-V delay occurred in the posterior SSS and was increased toward the anterior SSS (Fig. 3A). The mean distribution of voxel-wise pulse delay in the SSS progressively decreased with age (Fig. 3B). The distribution of voxel-wise pulse delay in the arteries did not show a strong trend with age (Supplemental Fig. 6).

### 3.4 Scan measurements were reproducible

**Fig. 4.**
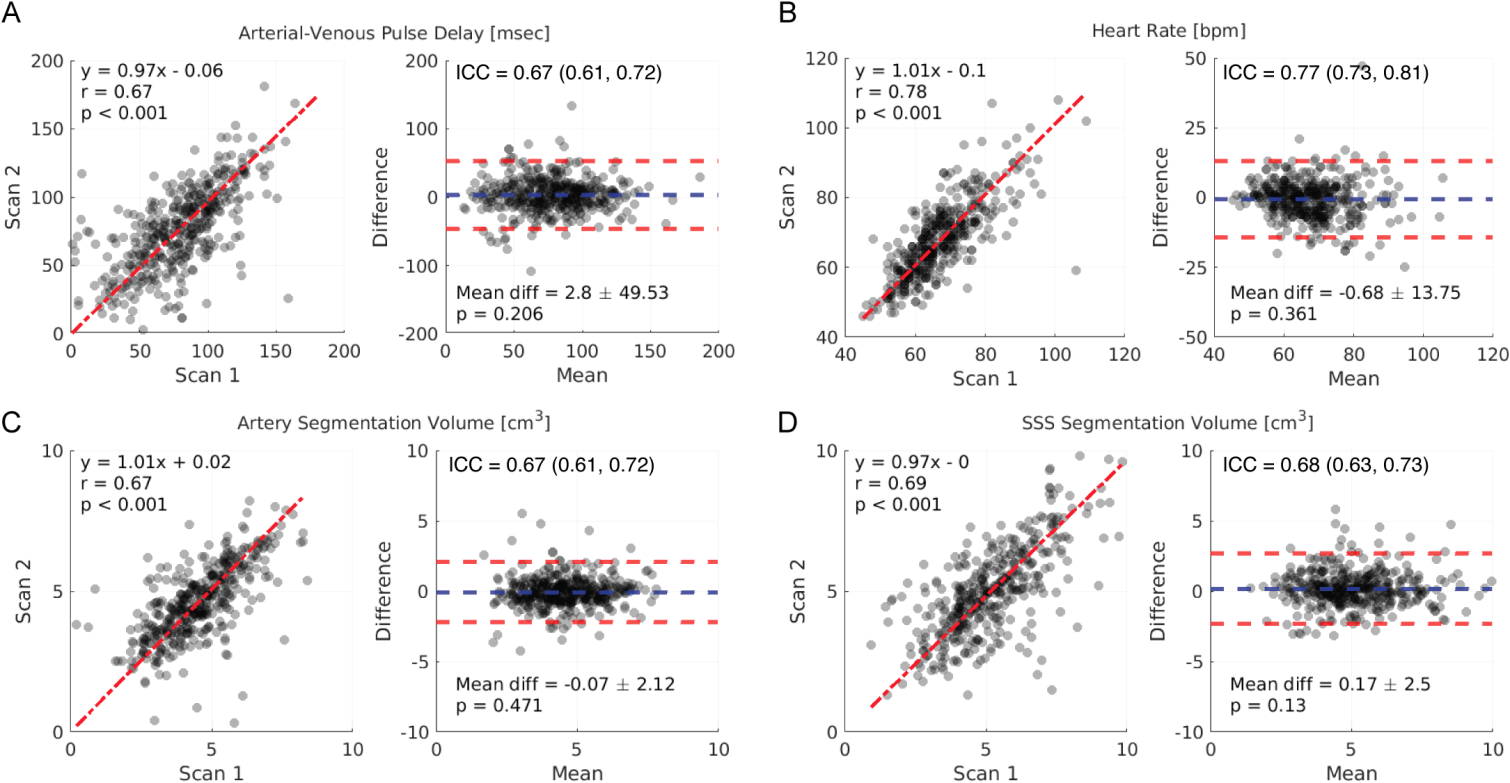
The arterial-venous (A-V) delay and other scan-specific measures were reproducible between scanning days (Left: Linear regression with total least-squares fit and Pearson correlation, red line: linear regression fit; Right: Bland-Altman plot with the difference calculated as Scan 1 minus Scan 2, blue line: mean difference, red lines: 95% confidence intervals of the difference). (A) A-V delay. (B) Scan heart rate. (C) Artery segmentation volume. (D) Superior sagittal sinus (SSS) segmentation volume. Abbreviations: A-V delay – arterial-venous delay, SSS – superior sagittal sinus, ICC – intraclass correlation coefficient.

The scan-specific measures were reproducible, including A-V delay, heart rate, artery segmentation volume, and SSS segmentation volume (Fig. 4). All measures demonstrated moderate to strong agreement between scans with intraclass correlation coefficients greater than 0.6. The direct comparison between scan measures is summarized in Figure 4; A-V delay measures had a strong correlation and agreement (y = 0.97x-0.06, r = 0.67, p < 0.001; mean diff = 2.8 ± 49.53 msec, p = 0.206), heart rate had a very strong correlation and agreement (y = 1.01-0.1, r = 0.78, p < 0.001; mean diff = -0.68 ± 13.75 beats per minute (bpm), p = 0.361), artery segmentation volume measures had a strong correlation and agreement (y = 1.01x+0.02, r = 0.67, p < 0.001; mean diff = -0.07 ± 2.12 cm^3^, p = 0.471), and SSS segmentation volume measures had a strong correlation and agreement (y = 0.97x-0.00, r = 0.69, p < 0.001; mean diff = 0.17 ± 2.50 cm^3^, p = 0.13).

### 3.5 fMRI A-V delay was consistent with and without cardiac aliasing

**Fig. 5.**
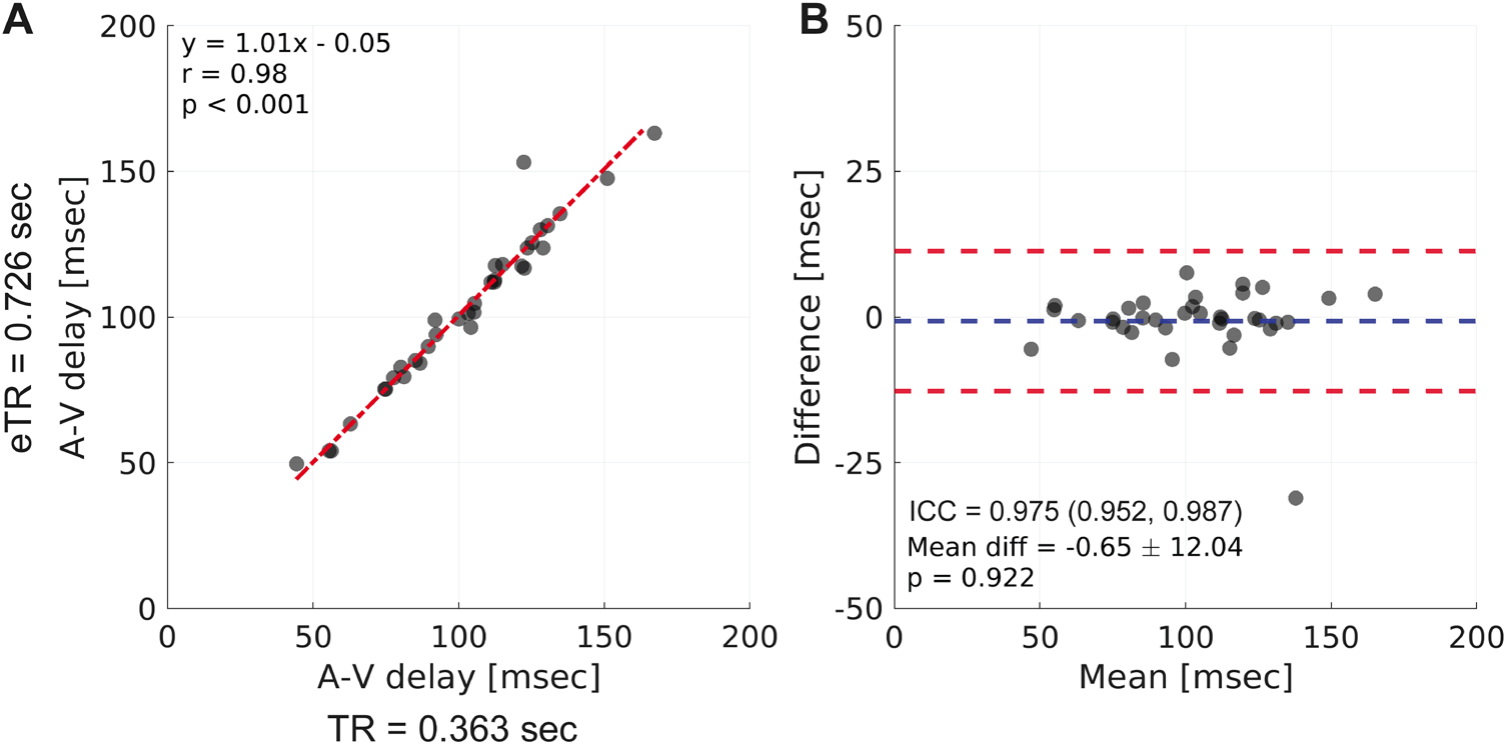
The fMRI arterial-venous (A-V) delay with and without cardiac aliasing showed strong agreement. Cardiac aliasing was absent in repetition time (TR) = 0.363 sec and present in effective TR (eTR) = 0.726 sec. (A) Linear regression with total least-squares fit and Pearson correlation demonstrated strong correlations between A-V delays calculated with TR and eTR (red line: linear regression fit). (B) Bland-Altman plot demonstrated strong agreement between A-V delays calculated with TR and eTR (difference calculated as the x-axis minus y-axis of the respective regression in A, blue line: mean difference, red lines: 95% confidence intervals of the difference) and a paired t-test between measured values (p > 0.05 indicated the measures were not different). Abbreviations: A-V delay – arterial-venous delay, repetition time – TR, effective TR – eTR, ICC – intraclass correlation coefficient.

The local cohort was used to compare the fMRI A-V delay with and without cardiac aliasing (see Supplemental Material for data inclusion criteria). The cohort consisted of 36 participants (28 women, 8 men), aged 53.3 ± 12.7 years (range: 35−82 years), each with a single scan. In the 36 analyzed scans, there was a strong correlation between the A-V delays calculated with TR = 0.363 sec and eTR = 0.726 sec (Fig. 5A, y = 1.01x - 0.05 msec, r = 0.98, p < 0.001). The A-V delays strongly agreed with a mean difference = -0.65 ± 12.04 msec and did not significantly differ between TRs (Fig. 5B, p = 0.922). The impact of cardiac aliasing on the fMRI signals, frequency responses, fMRI-PPG cross-correlations, and A-V delay calculations are summarized for a representative participant in Supplemental Figure 7.

## 4 Discussion

In this work, we developed a novel fMRI-based method to measure the A-V pulse delay, demonstrating the feasibility of using fMRI to assess the intracranial mechanical environment. By analyzing the HCP-A dataset, we uncovered a decrease in A-V delay with age and a shorter delay in men than in women. We observed a spatial pattern of the pulse delay within the SSS, providing insight into the passive compression of the SSS and intracranial pulse transmission. Finally, we validated that the A-V delay can be accurately measured in fMRI scans with cardiac aliasing.

The A-V delay differs from the arterial-to-venous blood transit time in both time duration and travel route. The blood transit time refers to the time required for blood to travel from the cerebral arteries, through the intermediate vessels, and into the cerebral venous sinuses. It is significantly longer (~5 sec (Liu et al., 2014; Yao et al., 2019)) than the A-V delay (~80 msec). While the blood transit route is confined to the vascular network, the route of pulsation contributing to the A-V delay does not transmit along the entire vascular network. Instead, arterial pulsations propagate through brain tissue and CSF, leading to venous sinus compression. The blood pulsatility of the venous sinus and the resulting A-V pulse delay represent a unique mechanical propagation exclusive to the brain, offering a unique perspective to assess the brain’s biomechanical properties.

Our studies’ fMRI-based A-V delay times were slightly shorter than previously published PC-MRI-derived measures. In this study, the mean A-V delay was 78.1 ± 32.1 msec (age: 58.8 ± 14.5 years) in healthy participants. Previous PC-MRI studies by Bateman et al. reported A-V delays in control participants of 89 ± 40 msec (age: 58 ± 7 years) (Bateman, 2000), 110 ± 70 msec (age: 42 ± 17 years) (Bateman, 2002), 112 ± 67 msec (age: 43 ± 16 years) and 111 ± 50 msec (age: 74 ± 16 years) (Bateman et al., 2016). Rivera-Rivera et al. have reported an A-V delay of 93 ± 50 msec (age: 71 ± 5 years, N = 68) (Rivera-Rivera et al., 2021). The mean fMRI-based A-V delay was slightly shorter than reported with PC-MRI; however, the range of A-V delays overlapped between the two methods. The slight deviations could be from differences in the location of the arterial pulse signal. The fMRI-based A-V delay was measured at the major cerebral arteries above the Circle of Willis. These major cerebral arteries are downstream of the internal carotid, vertebral, and basilar arteries, which are typically used in PC-MRI studies. The cardiac pulse enters the skull via the internal carotid and vertebral arteries, making them the earliest intracranial sites of arterial pulsation that drive SSS compression and venous blood pulsatility. Thus, the internal carotid and vertebral arteries are a more desirable site for measuring A-V delays, especially since the arterial pulse undergoes further damping before reaching the major cerebral arteries (Lefferts et al., 2020). In this work, we used the major cerebral arteries because their fMRI segmentations were more reliable. Future advancements in automatic fMRI segmentation of the internal carotid arteries may help address this limitation and increase agreement between fMRI- and PC-MRI-based A-V delays.

We showcased our method’s utility by studying the A-V delay in a large dataset, revealing a 4 msec reduction in A-V delay per decade of aging. This age-related reduction in A-V delay was not observed in previous PC-MRI studies with smaller sample sizes (highest N = 32 (Bateman et al., 2016; Stoquart-ElSankari et al., 2007)). The age-related decrease in A-V delay may be associated with well-documented aging processes that affect intracranial mechanical properties, including an increase in arterial stiffening (O’Rourke and Hashimoto, 2007; Zimmerman et al., 2021), an increase in CSF outflow resistance (Albeck et al., 1998; Czosnyka et al., 2001), and a reduction in brain volume (Fujita et al., 2023). An increase in arterial stiffness leads to faster pulse propagation (increased pulse wave velocity) of the arterial pressure wave along the vascular tree (Björnfot et al., 2021; Wentland et al., 2014). CSF outflow resistance refers to the difficulty of CSF flowing from the cranium to the spinal canal, which increases with age (Albeck et al., 1998; Czosnyka et al., 2001). Increased CSF outflow resistance reduces intracranial compliance (Wagshul et al., 2011), meaning the intracranial capacity to compensate for volume changes decreases. This reduced compliance contributes to faster cardiac pulse propagation. Lastly, reduced brain volume increases the ratio of CSF to brain tissue, increasing the proportion of incompressible CSF within the intracranial space and enhancing the efficiency of pulse transmission. These combined age-related biomechanical changes are all plausible explanations for the observed decrease in A-V delay.

In our analysis, the A-V delay was 12 msec shorter in men than in women. Similar to the reduction of A-V delay with age, the reduction in men could be due to increases in arterial stiffness, which have been reported to be higher in men (Doonan et al., 2013). Studies have not yet assessed the differences in CSF outflow resistance between men and women (Albeck et al., 1998; Czosnyka et al., 2001; Eklund et al., 2007). Finally, we observed that men had a lower normalized brain volume (brain-to-skull ratio, Supplemental Fig. 8), demonstrating an increased CSF to brain tissue ratio. A combination of higher arterial stiffness and lower normalized brain volume could contribute to the lower A-V delay observed in men.

There was a significant negative relationship between A-V delay and heart rate, indicating that the A-V delay shortens as the heart rate increases. This finding suggests that pulse propagation speed may increase with heart rate. The pulse wave contributing to the A-V delay propagates along the major arteries and through the CSF to compress the SSS. Our findings align with literature showing that pulse wave velocity increases with heart rate along the arterial and CSF pathways. Along the arterial pathway, previous studies have reported a positive relationship between pulse wave velocity and heart rate in the central (aortic-femoral) (Papaioannou et al., 2019) and cerebral arteries (Rivera-Rivera et al., 2024). Along the CSF pathway, transient increases in heart rate are closely linked to increases in intracranial pressure (Dimitri et al., 2017), which will increase CSF pressure and pulse wave speed through CSF. Therefore, increased propagation speed through arterial and CSF routes with higher heart rates likely shortens the A-V delay.

The group-averaged A-V delay maps illustrated a clear delay pattern in the SSS. A shorter delay was observed in the posterior SSS, and a longer delay was observed toward the anterior SSS. The SSS voxel position partly explained the bimodal distribution of A-V delays, where short delays were most prominent in the posterior SSS and longer delays in the anterior SSS (Supplemental Fig. 9, Supplemental Video 1). The pulse delay pattern is opposite of the anterior to posterior blood flow direction in the SSS (Brisman et al., 1972). Studies explicitly investigating cerebral venous pulsations suggest that pulsations occur first in larger venous sinuses and later in smaller cortical veins (Driver et al., 2020). Our results complement these findings and provide another viewpoint on cardiac pulse transmission in cerebral veins. They support that the cardiac pulse causes an early compression of the posterior SSS, followed by a later compression of the anterior SSS and smaller cortical veins, progressing in a pattern opposite to the direction of venous blood flow. Our method enables the measurement of A-V delay in fMRI scans with typical time resolutions, opening new opportunities to study its alterations in disease.

A few aspects related to A-V delay calculation warrant caution in future studies. First, the cross-correlation analysis involves aligning slice timing with the PPG signal, which precludes motion correction for fMRI data as it would disrupt slice timing in interleaved slice acquisitions. Second, the proposed approach depends heavily on the quality of the PPG signal. Approximately 20% of the data from HCP-A were excluded due to poor PPG signal quality, likely due to less emphasis on this physiological component during data acquisition, as it was not a central aspect of the study design. Ensuring a good PPG connection during scanning can significantly improve signal quality. Third, it is essential to note that A-V delay represents a time delay and is a surrogate measure of intracranial compliance. The computation of intracranial compliance requires measuring fluid volume changes (Alperin et al., 2000). This study completed the measures in a supine orientation, and the postural dependence of A-V delay could not be explored (Alperin et al., 2021). Fourth, we excluded negative A-V delays in this analysis as it is physiologically implausible for venous pulsation to precede arterial pulsation. It is unclear whether these errors originate from borderline PPG signal quality or scan motion that narrowly met quality control thresholds and warrants further investigation. A-V delays near zero may contain similar measurement errors, though such values have been reported in previous PC-MRI studies (Bhadelia et al., 1997; Rivera-Rivera et al., 2021). Lastly, this analysis was retrospective; a longitudinal study is necessary to validate the dependence of A-V delay on age.

To conclude, this work demonstrates the feasibility of extracting A-V delay and assessing the intracranial mechanical environment using fMRI, highlighting a previously unexplored capability of this imaging modality. The fast scan protocol and fully automated processing make it an accessible tool, broadening fMRI’s potential applications by adding a biomechanical dimension to its established roles in evaluating neuronal and hemodynamic function. This innovative approach uncovered that A-V delay decreased with age and was shorter in men than women. Given the widespread availability of fMRI, this method holds promise for future studies investigating biomechanical changes associated with aging and disease.

## Supplementary Material

Supplementary Material

Supplementary Video

## Data Availability

The data came from the HCP-Aging 2.0 Release, DOI: 10.15154/1520707. The study data is available for download through the NIMH Data Archive (https://nda.nih.gov/). The local cohort data used in this manuscript will be available upon reasonable request due to privacy/ethical restrictions. The code will be made available upon reasonable request.
