# Supplementary material for: An fMRI approach to assess intracranial arterial-to-venous cardiac pulse delay in aging
Source: Imaging Neurosci (Camb). 2025 Oct 30;3:IMAG.a.969. doi: 10.1162/IMAG.a.969 (PMC12576844; doi:10.1162/IMAG.a.969)
Supplement: Supplementary Material [file IMAG.a.969_supp.pdf]

# Supplemental Material:

## Methods:

The arterial-venous (A-V) delay can be calculated by taking the difference between arterial and venous pulse arrivals relative to the finger pulse. The relative arrival times are defined as the TimeDelay. A summary of the cardiac pulsation timeline in the body and derivation/reasoning behind the fMRI A-V delay calculation is provided in Supplemental Figure 1.

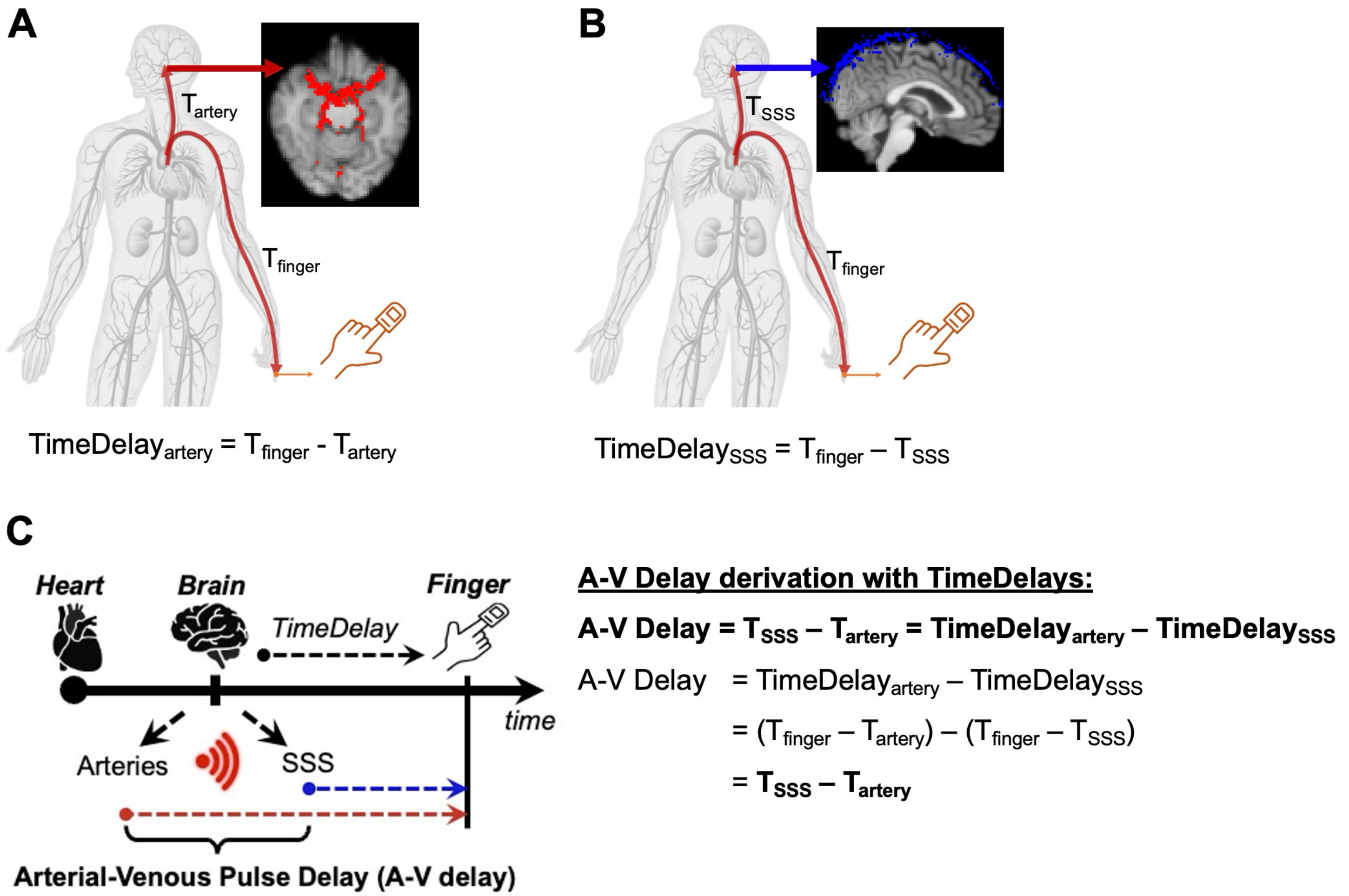


***Supplemental Figure 1:*** *The intuition behind the fMRI arterial-venous (A-V) pulse delay calculation.* ***A.*** *The TimeDelay calculation of the arteries.* ***B.*** *The TimeDelay calculation of the superior sagittal sinus (SSS).* ***C.*** *The timeline of the cardiac pulsation from the heart to the arteries, SSS, and finger.* ***D.*** *The intuition of why the difference in TimeDelay between the arteries and SSS is equivalent to the A-V delay.*

Supplemental Figure 2 summarizes the fMRI–finger PPG cross-correlation curves for the anterior, middle, and posterior cerebral arteries (ACA, MCA, PCA), as well as the superior sagittal sinus (SSS) for a representative participant. The minimum signal, corresponding to the TimeDelay between the brain and finger cardiac pulse arrival, was consistent between arterial segments.

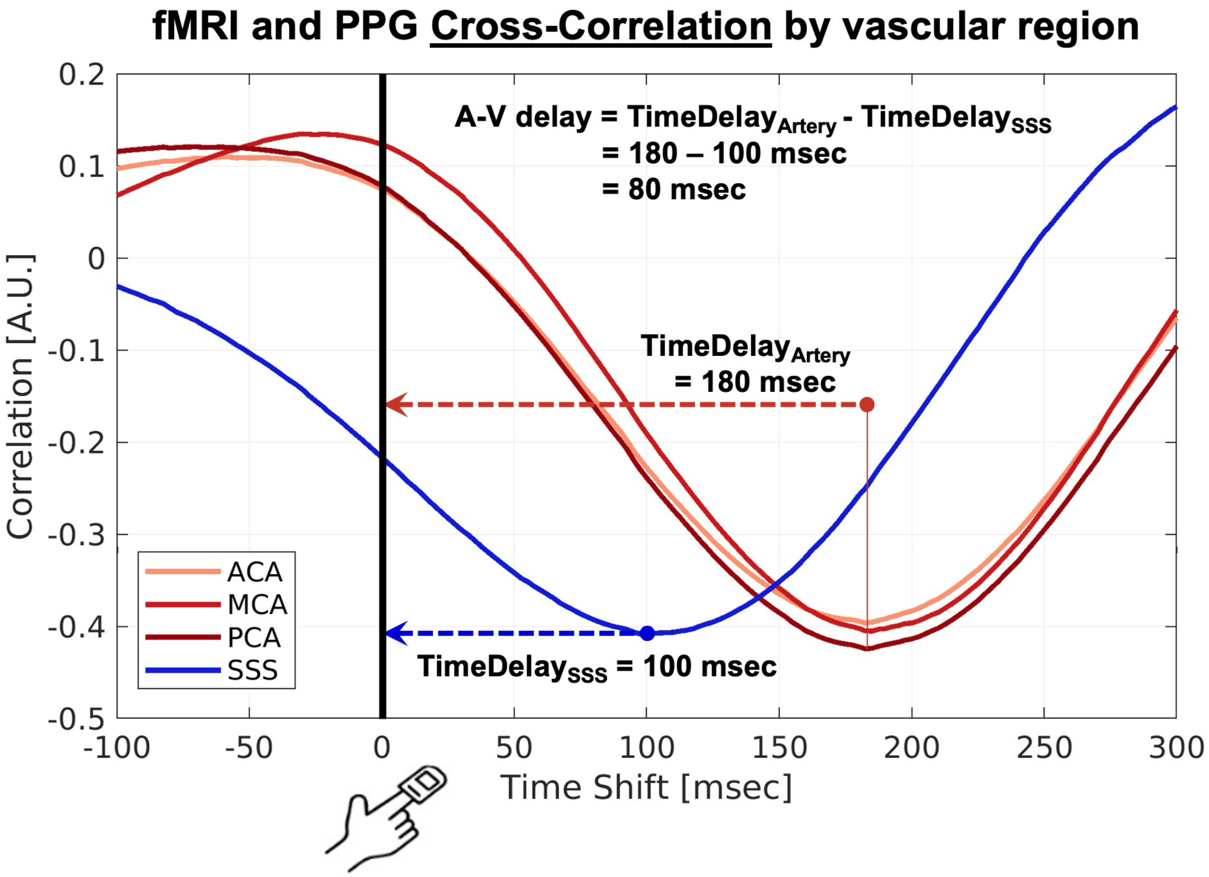


***Supplemental Figure 2:*** *A representative participant fMRI-finger cross-correlation separated by major vascular compartment, including the anterior, middle, and posterior cerebral arteries (ACA, MCA, PCA) as well as the superior sagittal sinus (SSS).* ***Abbreviations:*** *ACA – anterior cerebral artery, MCA – middle cerebral artery, PCA – posterior cerebral artery,* SSS – superior sagittal sinus, PPG – photoplethysmography.

The window size of the cross-correlation between finger PPG and voxel-wise fMRI signal was set to ±300 msec to reduce computational expense while accurately identifying the time delay of interest. Supplemental Figure 3 illustrates the mean ± standard deviation of the cross-correlation in the superior sagittal sinus (SSS, blue) and artery (red) regions of interest for a representative participant. The strongest negative correlation corresponding to the TimeDelay aligns with findings from other studies, which demonstrate a negative fMRI signal corresponding to the cardiac pulse arrival (Hermes et al., 2023; Rajna et al., 2021). Over the longer time window, it can be observed that the neighboring negative troughs are smaller in magnitude than the initial identified trough (dashed line in Supplemental Figure 3A).

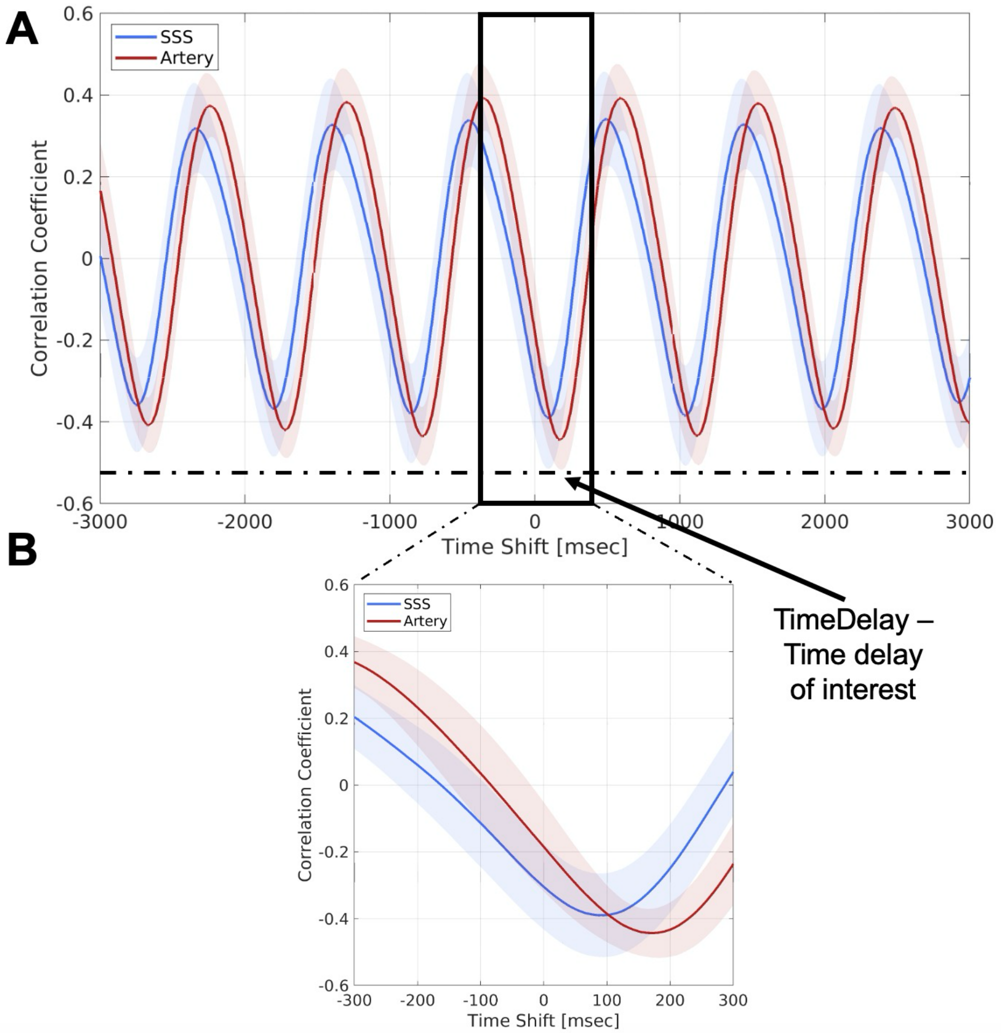


***Supplemental Figure 3:*** *Window size comparison of the cross-correlation between finger PPG and voxel-wise fMRI signal in the superior sagittal sinus (SSS) and artery region of interest. The solid line is the voxel-wise mean and shaded region is the standard deviation of a representative participant.* ***A.*** *Window size of ±3000 msec.* ***B.*** *Window size of ±300 msec.* ***Abbreviations:*** SSS – superior sagittal sinus.

All the covariates in the initial linear mixed-effects model are included in Supplemental Table 1. The Race covariate included White, Black, Asian, American Indian, More than once race, and Other. The Ethnicity was Not Latino or Latino.

***Supplemental Table 1:*** *Covariates included in the linear mixed-effects model.*

| **Demographics** | **Brain Volume Measures** | **Blood Tests** |
|---|---|---|
| Age | Cortex | Estradiol |
| Biological Sex | Total Gray Matter | Follicle Stimulating Hormone |
| Race | Subcortical Gray Matter | Luteinizing Hormone |
| Ethnicity | Cerebral White Matter | Vitamin D |
| **Ambulatory Measures** | **Blood Tests** | Glucose |
| Scan Heart Rate | Fasted Blood (Yes/No) | Insulin |
| Systolic Blood Pressure | Alanine transaminase (ALT) | Hemoglobin A1c |
| Diastolic Blood Pressure | Alkaline phosphatase (ALP) | Total Protein |
| Cognitive Score - MoCA | Aspartate aminotransferase (AST) | Albumin |
| Years of Education | Calcium | Bilirubin |
| **Brain Volume Measures** | Chloride | Total Cholesterol |
| Large Cerebral Artery | Creatinine | Low-Density Lipoprotein |
| Superior Sagittal Sinus | Potassium | High-Density Lipoprotein |
| Normalized Brain | Sodium | Total Triglycerides |
| Estimated Total Intracranial | Urea | CO2 Content |
| Lateral Ventricle | Testosterone | C-reactive protein |

## Results:

The relationship between arterial-venous (A-V) delay and segmentation volumes are summarized in Supplemental Figure 4. The superior sagittal sinus volume was significantly associated with the A-V delay ($\beta$=3.358 msec/cm$^3$, SE=0.683, p<0.001). The artery volume was excluded from the model because it was not significantly associated with A-V delay and did not improve the model.

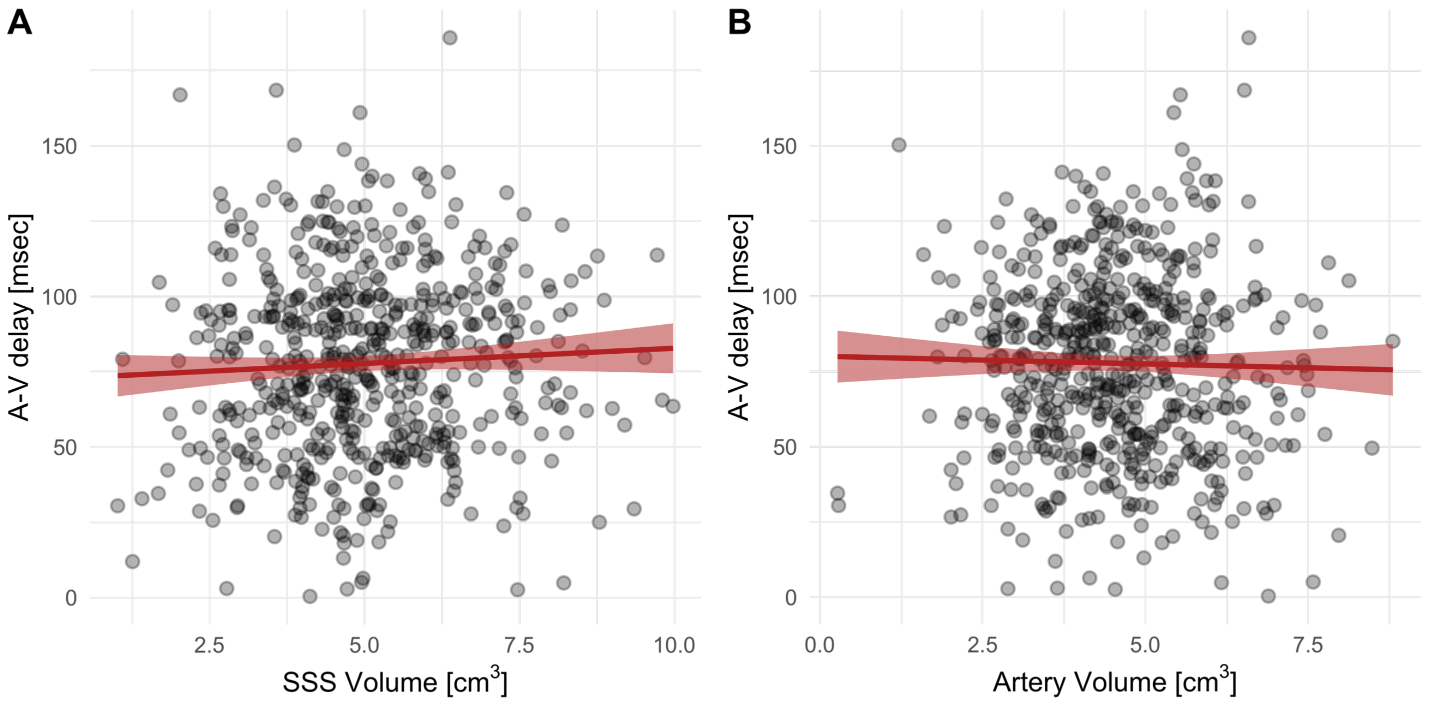


***Supplemental Figure 4:*** *The relationship between arterial-venous (A-V) delay and* ***A.*** *superior sagittal sinus (SSS) volume and* ***B.*** *artery volume.* ***Abbreviations:*** *A-V delay – arterial-venous delay*, SSS – superior sagittal sinus.

Post hoc, we tested if the interaction between age and biological sex improved the A-V delay model and whether there was a relationship between the interaction term and the A-V delay. After adding the interaction term to the final model, there was no significant model fit improvement ($\chi^2(1)$ = 1.15, p = 0.284), and the interaction term was not significantly correlated with the A-V delay (t=-1.06, p = 0.288). We excluded the interaction term from the final model based on these results.

The quantile-quantile (Q-Q) plot for the best-fit linear mixed effects model is summarized in Supplemental Figure 5. The heavy tails might indicate that the standard error estimates are slightly larger than the final model calculated. Still, it suggests that the model residuals do not heavily deviate from a normal distribution.

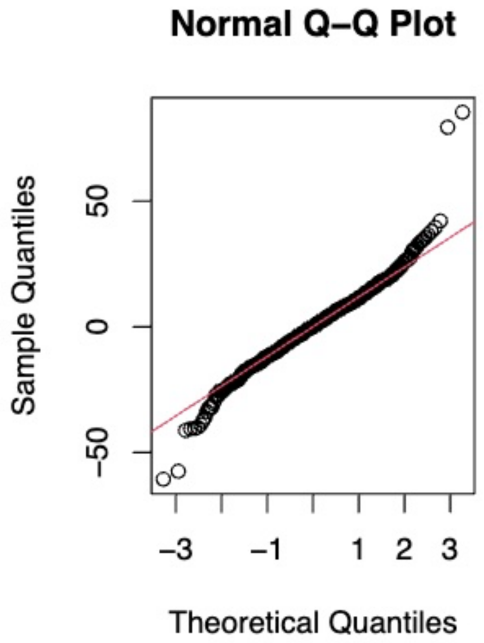


***Supplemental Figure 5*:** The quantile-quantile (Q-Q) plot for the best-fit linear mixed effects model.

Four age groups were used to create group-averaged vascular pulse delay maps in MNI space. The age demographics and number of participants in each group are summarized in Supplemental Table 2. Each group consisted of around 100 participants.

**Supplemental Table 2:** *Age group demographics for the group-averaged vascular pulse delay MNI space maps.*

**Age by Group [years]**

| | Mean | Standard Deviation | Min | Max | N | Men/Women |
|---|---|---|---|---|---|---|
| **Group 1** | 41.4 | 3.1 | 36.2 | 47.0 | 100 | 44/56 |
| **Group 2** | 52.2 | 3.0 | 47.1 | 56.9 | 102 | 38/64 |
| **Group 3** | 63.2 | 3.8 | 57.1 | 69.8 | 109 | 47/62 |
| **Group 4** | 78.1 | 4.9 | 70.1 | 88.5 | 103 | 49/54 |

The distribution of artery pulse delay with respect to the middle cerebral artery (MCA) did not have a strong trend between age-groups (Supplemental Figure 6).

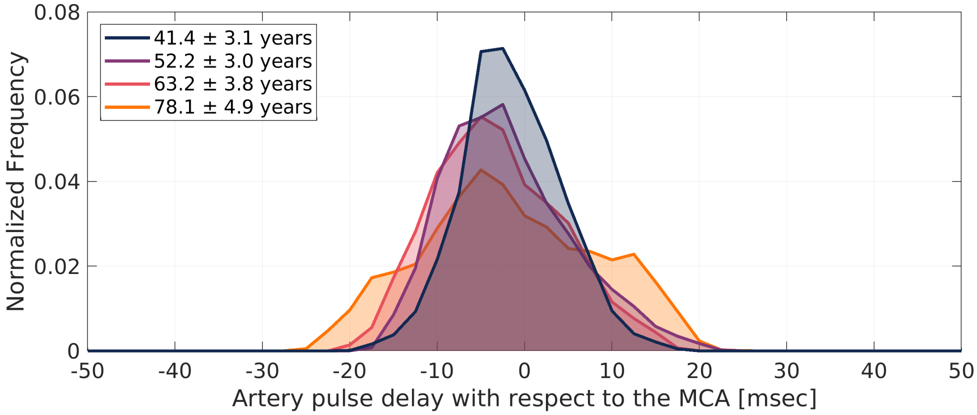


***Supplemental Figure 6:*** *Artery pulse delay with respect to the middle cerebral artery (MCA) by age group.*

The local cohort was used to compare the fMRI A-V delay calculation with and without cardiac aliasing. The local cohort included 46 participants. Of the 46 scans, 9 were excluded because of data quality concerns (6 exhibited high motion, and 3 had poor PPG quality). Additionally, one scan was excluded because the heart rate was greater than 82 beats per minute, which resulted in aliased cardiac activity at TR = 0.363 sec.

The impact of cardiac aliasing on the raw fMRI signal, power spectral density, fMRI-PPG cross-correlations, and A-V delay measurement are summarized for a representative participant in Supplemental Figure 7. Panels A-C were generated with the native TR = 0.363 sec, where the cardiac signal is critically sampled. Panels D-E were generated with an effective TR (eTR) = 0.726 sec, where the cardiac signal is aliased.

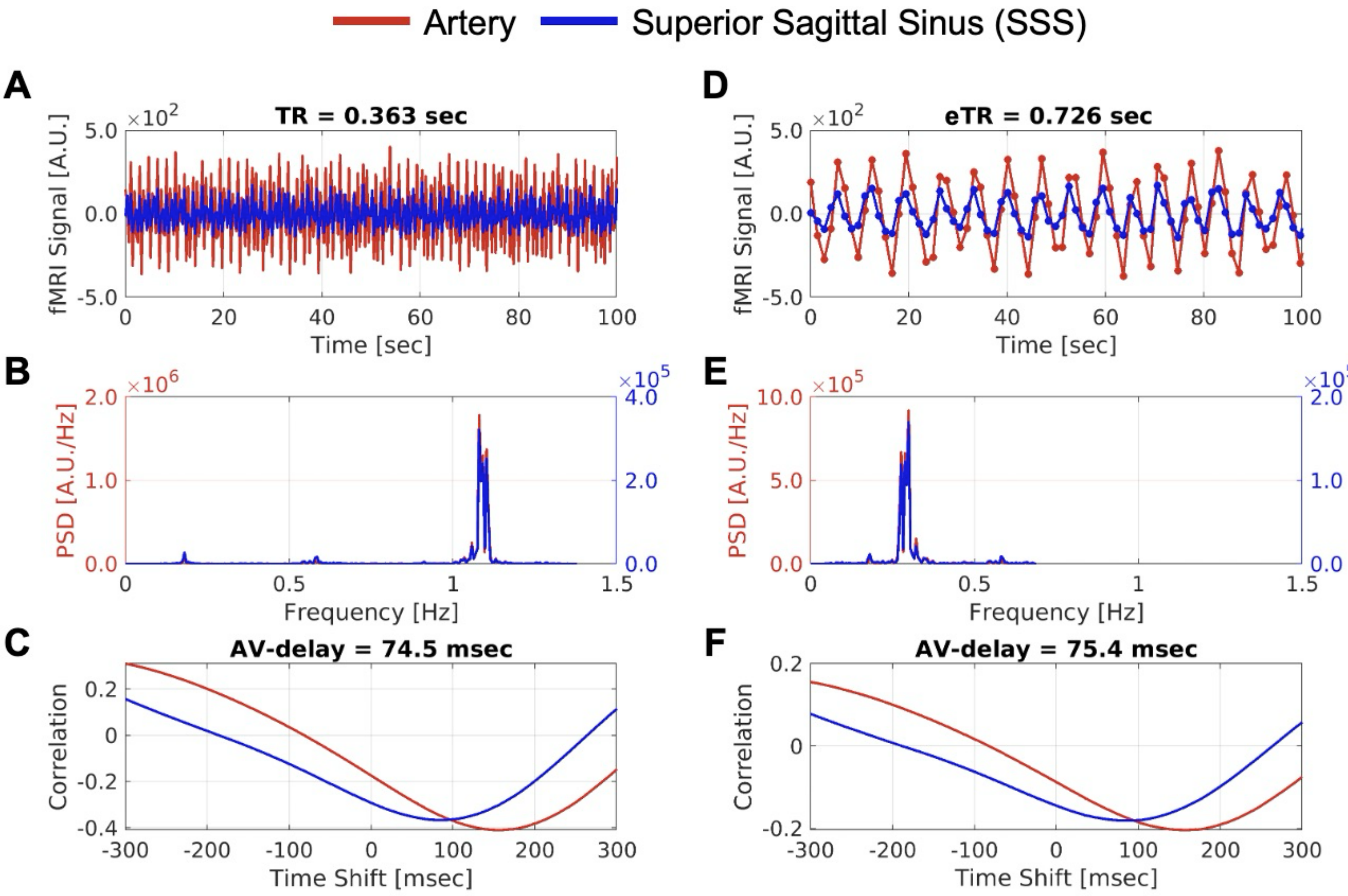


***Supplemental Figure 7:*** *The impact of cardiac aliasing on the raw fMRI signal **(A & D)**, power spectral density (PSD, **B & E**), cross-correlations **(C & F)**, and arterial-venous (A-V) delay without (left) and with (right) cardiac aliasing. Panels **A-C** summarize repetition time (TR) = 0.363 sec, where the cardiac signals are critically sampled. Panels **D-F** summarize an effective TR (eTR) = 0.726 sec, where the cardiac signals are aliased.*

## Discussion:

The normalized brain volume was lower in men (71.2 ± 5.8 %) than in women (74.4 ± 8.1 %), as shown in Supplemental Figure 8 (two-sample t-test, t = 5.31, p<0.001, 95% CI of difference = (2.02, 4.40%)).

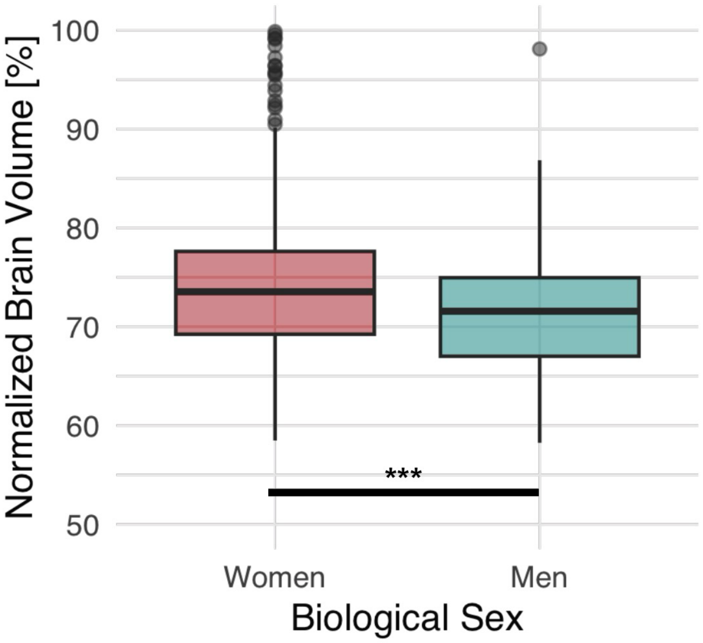


***Supplemental Figure 8:*** *Relationship between normalized brain volume and biological sex. ***p<0.001.*

*The bimodal distribution of SSS pulse delay times with respect to the arterial arrival was related to the SSS voxel's posterior-to-anterior position.*

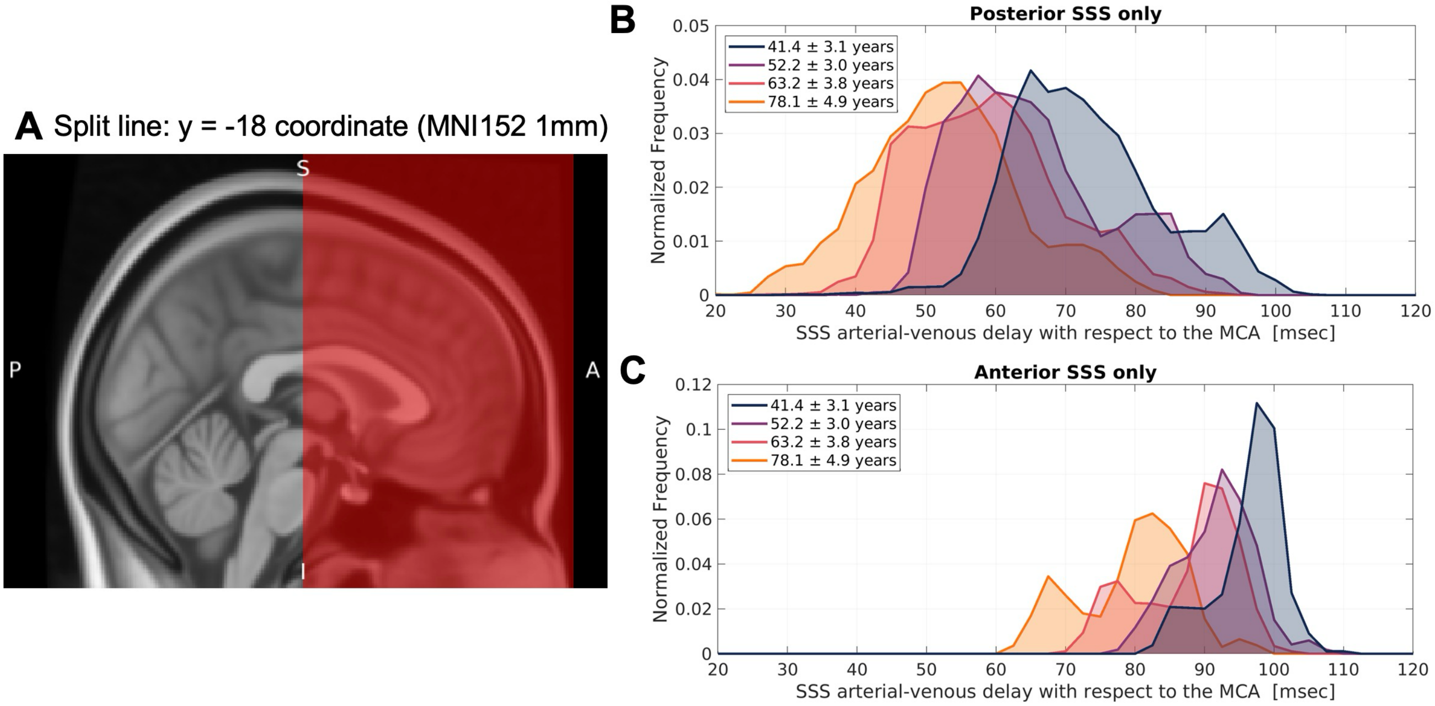


***Supplemental Figure 9:*** *Summary of the superior sagittal sinus (SSS) pulse delay with respect to the middle cerebral artery (MCA) stratified by posterior-to-anterior position.* ***(A)*** *The MNI152 1 mm standard brain was divided into anterior and posterior regions. SSS voxels with y-coordinates from coordinates -126 to -18 were classified as posterior, while -17 to 90 were classified as anterior. The frequency distributions of SSS pulse delays between age groups were normalized to have an integral of one for both the* ***(B)*** *posterior SSS and* ***(C)*** *anterior SSS.*

## References:

Hermes, D., Wu, H., Kerr, A.B., Wandell, B.A., 2023. Measuring brain beats: Cardiac-aligned fast functional magnetic resonance imaging signals. Hum Brain Mapp 44, 280–294. https://doi.org/10.1002/HBM.26128

Rajna, Z., Mattila, H., Huotari, N., Tuovinen, T., Krüger, J., Holst, S.C., Korhonen, V., Remes, A.M., Seppänen, T., Hennig, J., Nedergaard, M., Kiviniemi, V., 2021. Cardiovascular brain impulses in Alzheimer's disease. Brain 144. https://doi.org/10.1093/brain/awab144
